# Lanzhang Granules Ameliorate Nonalcoholic Fatty Liver Disease by Regulating the PPAR*α* Signaling Pathway

**DOI:** 10.1155/2022/1124901

**Published:** 2022-01-07

**Authors:** Ping Huang, Lili Yang, Yang Liu, Yuwei Jiang, Yiping Li, Zhiwei Chen, Haiyan Song, Peiyong Zheng

**Affiliations:** ^1^Institute of Digestive Diseases, Longhua Hospital, Shanghai University of Traditional Chinese Medicine, Shanghai, China; ^2^Teaching Experiment Center, Shanghai University of Traditional Chinese Medicine, 201203 Shanghai, China

## Abstract

**Background:**

There is still a lack of effective therapeutic drugs for nonalcoholic fatty liver disease (NAFLD) to date. In this study, we applied mouse model experiments to clarify the effect of Chinese herbal medicine “Lanzhang Granules (LZG)” on NAFLD and further explore the potential mechanism to provide an alternative method for NAFLD treatment.

**Methods:**

Male C57BL/6J mice were fed with a high-fat diet (HFD) for twenty-two weeks to induce the NAFLD model. LZG intervention was then performed by gavage daily for another eight weeks. At the end of the treatment, serum and liver tissues were collected. Serum biochemical indexes, insulin levels, and liver histopathology were measured to assess the effect of LZG on NAFLD. The liver tissues were then analyzed by RNA sequence for differentially expressed genes and signaling pathways. Results were further analyzed by Protein-Protein Interaction (PPI) networks between the LZG and model groups. The selected different genes and signaling pathways were further verified by RT-PCR and Western blot analysis. Moreover, alpha mouse liver 12 (AML12) cells with lipid accumulation induced by fatty acid were treated with LZG, Fenofibrate (PPAR*α* agonist), or Gw6471 (PPAR*α* antagonist) to confirm the potential pharmacological mechanism.

**Results:**

LZG was found to downregulate liver weight, body weight, liver index, and serum levels of ALT, AST, and serum lipid in HFD-induced NAFLD mice. HE and Oil Red O staining showed the improvement of hepatic steatosis and inflammatory infiltration in the mice with LZG treatment. The homeostasis model assessment-insulin resistance (HOMA-IR) index indicated that LZG improved the insulin resistance of NAFLD mice. The RNA sequencing and PPI analysis confirmed the role of LZG in lipid metabolism regulation and identified the peroxisome proliferator-activated receptor alpha (PPAR*α*) signaling pathway as one of the major underlying mechanisms. Western blot and RT-PCR results verified the regulatory effect of LZG on the PPAR*α* pathway, including the upregulation of PPAR*α*, acyl-coenzyme A oxidase 1 (ACOX1), and enoyl-CoA hydratase and 3-hydroxyacyl-CoA dehydrogenase (EHHADH) and the downregulation of TNF*α*. *In vitro* experiments showed the effect of LZG in improving lipid accumulation and cell viability in AML12 cells induced by fatty acids, which were alleviated by Gw6471 coincubation. Gw6471could also reverse the transcription of PPAR target genes ACOX1 and EHHADH, which were upregulated by LZG treatment.

**Conclusion:**

LZG can improve NAFLD in mice or cell models. A major underlying mechanism may be the regulation of the PPAR*α* signaling pathway to improve lipid metabolism and inhibit the inflammatory response. This study will help to promote the clinical application of LZG for the treatment of NAFLD.

## 1. Introduction

Nonalcoholic fatty liver disease (NAFLD) is a chronic liver injury disease that is associated with lipid accumulation and insulin resistance. In 2017, the global prevalence of NAFLD was approximately 24%. With changes in daily lifestyle and dietary habits, the incidence of NAFLD continues to rapidly increase throughout the world. Without proper and effective treatment, some cases of NAFLD could progress to nonalcoholic steatohepatitis (NASH), cirrhosis, and hepatocellular carcinoma [1, 2]. Studies have found that NAFLD accounts for 14.1% of the causative factors of hepatocellular carcinoma and is increasing annually at a rate of 9% [3]. NAFLD treatment has previously focused on lifestyle changes and pharmacological treatment for clinical manifestations and liver injury. However, there is still lack of drugs for NAFLD treatment proven to be effective in clinical trials [4].

In recent years, traditional Chinese medicine (TCM) has been widely used and shown efficacy in the treatment of NAFLD in China. In clinical application, many TCM formulas have been reported to have effects on NAFLD by improving TCM syndrome, serum lipid profile, liver function, and B ultrasonic index [5]. According to TCM theory, NAFLD is mainly caused by excessive consumption of greasy and sweet food, emotional disorders, excessive labor or leisure, and congenital deficiencies. The pathogenesis of NAFLD is mainly spleen qi deficiency or spleen yang deficiency. The spleen dysfunction leads to phlegm and dampness in the liver with stagnant qi and blood, which results in chronic liver injury [6, 7]. Based on the aforementioned TCM theory, we have developed the formula “Lanzhang Granules (LZG),” which consists of the following herbal medicines: *Gynostemma pentaphyllum (*Thunb.) Mak, *Astragalus mongholicus, Radix Angelicae Sinensis*, *Radix et Rhizoma Tigrinum*, and *Fritillariae Thunbergii Bulbus.* While this formula has shown to be beneficial in the clinical treatment of NAFLD, the efficacy still lacks experimental evidence, and the underlying mechanism remains unclear.

In this study, we applied a high-fat diet (HFD) induced NAFLD C57BL/6J mice model and intervened with LZG to observe its efficacy. To further explore the pharmacological mechanism of LZG, the differential genes and related signaling pathways were screened by genomics analysis of liver tissues of mice both with and without LZG intervention. Further experiments *in vivo* and *in vitro* were carried out to verify the potential pharmacological mechanism. The results found that LZG was effective in the NAFLD treatment and that the PPAR*α* signaling pathway might be the major mechanism for this effect. This study will provide a scientific basis for NAFLD treatment with LZG and promote its application in clinical practice.

## 2. Materials and Methods

### 2.1. Experimental Animals and Design

C57BL/6J male mice were purchased from Shanghai SLAC Laboratory Animal Co., Ltd. (Production License No. SCXK (Shanghai) 2017–0005). The mice were randomly divided into the control group (*n* = 6) and the model group (*n* = 12) according to their body weight. The normal control group was given a chow diet, and the model group was given a 60% high-fat diet (Research Diet, Inc., Rodent Diet with 60% kcal from fat, D12492) diet. After twenty-two weeks, the model group was redivided, according to body weight, into the model group and the LZG group (*n* = 6 per group). Mice in the LZG group were given a continuous gavage of LZG. Body weight and food intake were measured weekly. Eight weeks later, the mice fasted for 12 hours. After mice were anesthetized, blood was collected from the retroorbital sinus, and serum was separated through centrifugation and cryopreserved. Then, mice were humanely sacrificed. Liver tissues were frozen immediately or fixed in 4% neutral paraformaldehyde for later measurement. The experimental procedures were designed following the guidelines for the feeding and use of experimental animals and approved by the Animal Experiment Ethics Committee of Longhua Hospital, Shanghai University of Traditional Chinese Medicine (LHERWA-190001).

### 2.2. Experimental Drugs

The formula of LZG consisted of five raw herbal medicines: 30 g of *Gynostemma pentaphyllum* (Thunb.) Mak, 30 g of *Astragalus mongholicus*, 15 g of *Radix Angelicae Sinensis*, 20 g of *Radix et Rhizoma Tigrinum*, and 9 g of *Fritillariae Thunbergii Bulbus*. The extract from raw herbs (Sichuan Neo-Green Pharmaceutical Technology Development Co., Chengdu) was used in this study, with lot numbers: *Gynostemma pentaphyllum (Thunb.) Mak* (lot: 19030064), *Astragalus mongholicus* (Lot: 19030203), *Radix Angelicae Sinensis* (lot: 19030121), *Radix et Rhizoma Tigrinum* (lot: 19090225), and *Fritillariae Thunbergii Bulbus* (lot: 19030001). Each drug was converted according to the yield rate and then mixed into the LZG compound, which was diluted to the desired concentrations in distilled water for oral gavage or cell incubation. The intervention dose of LZG for mice (1.6 g/kg body weight once a day) was calculated according to the equivalent dose in humans.

### 2.3. Measurement of Serum Transaminase, Lipid, and Tumor Necrosis Factor-*α* (TNF*α*) Levels

The ROCHE cobas 8000 modular analyzer series and corresponding reagent kits (Basel, Switzerland) were used to detect serum alanine aminotransferase (ALT), serum aspartate aminotransferase (AST), lactate dehydrogenase (LDH), triglycerides (TG), total cholesterol (TC), high-density lipoprotein cholesterol (HDL-c), and low-density lipoprotein cholesterol (LDL-c).

Serum TNF*α* level was measured using an ELISA kit (Shanghai Westang Biotechnology). The Synergy H4 Hybrid Multi-Mode microplate reader (BioTeck, Winooski, USA) was used to detect the absorbance value at 450 nm, and the content of TNF*α* was calculated according to the product manual.

### 2.4. Homeostasis Model Assessment-Insulin Resistance (HOMA-IR) Index

Fasting plasma glucose (FPG) in mice was measured by ROCHE cobas 8000 modular analyzers. The level of fasting insulin (FIN) was measured by the ELISA method. The ELISA kit was purchased from Shanghai Westang Biotechnology. The following formula was applied to calculate the HOMA-IR value:

(1) $$\begin{array}{c} \text{HOMA}-\text{IR}=\frac{\text{FPG}*\text{FIN}}{22.5}. \end{array}$$

### 2.5. Hepatic Tissue Hematoxylin-Eosin (HE) Staining and Oil Red O Staining

The liver tissue was fixed in 4% neutral paraformaldehyde for 48 hours. After this, samples were washed in running water for 6 hours. They were then dehydrated, paraffin-embedded, sectioned at 5 *μ*m per slice, oven-dried at 60°C for 2 hours, dewaxed, and processed for HE staining. Samples were then sealed and photographed under microscope observation.

Liver tissues were fixed in 4% neutral paraformaldehyde for 4 hours, placed in 30% sucrose water at 4°C, thoroughly dehydrated, and then frozen at −80°C. Frozen liver tissues were then coated with OCT (Sakura, Tokyo, Japan) and sectioned at 10 µm. After coating, the section was then stained with Oil Red O (Sigma) and counterstained with hematoxylin. Samples were observed and photographed under the microscope to assess lipid accumulation in liver tissues.

### 2.6. Determination of TG and TC of Liver Tissues

Liver tissues were added to anhydrous ethanol at the ratio of 1 : 9 weight to volume (g : mL). Samples were homogenized and centrifuged at 2500 rpm for 10 minutes. Then, the supernatant was extracted. TG and TC assay kits (Nanjing Jiancheng Biological Engineering Research Institute, Nanjing, China) were used to detect liver lipids using the GPO-PAP enzyme method. The samples were then added and incubated at 37°C for 10 minutes. The absorbance was then measured at 510 nm by applying a microplate detector Synergy H4 Hybrid Reader (BioTek, USA), and calculations were performed.

### 2.7. RNA Sequencing Analysis of Liver Tissues

Total RNA extraction of samples was performed using RNAiso Plus Total RNA extraction reagent (TAKARA) and according to the standard procedure provided by the manufacturer. The RNA Clean XP Kit (Beckman Coulter, Inc., Kraemer Boulevard Brea, CA, USA) and RNase-Free DNase Set (QIAGEN, GmBH, Germany) were used to purify total RNA. Libraries were constructed and completed using cluster generation and hybridization of first-order sequencing primers. Sequencing reagents were then prepared according to the Illumina User Guide, and flow cells with clusters were loaded onto the machine. The paired-end program was selected to perform double-end sequencing, which was controlled by the data collection software provided by Illumina. Then, real-time data analysis was performed. Gene expression was measured using fragments per kilobase of exon model per Million (FPKM) mapped reading values, and the corrected *p* value values were used to indicate the different significance between samples. Fold change (FC) values were measured to indicate the fold of difference. We screened for differential genes based on *q* value ≤0.05 and fold change ≥2.

### 2.8. Protein Correlation Analysis by Protein-Protein Interaction (PPI) Networks

The 13 significant differential genes that were previously screened out were entered into the PPI database (https://string-db.org/cgi/input.pl). Then, protein interaction networks were constructed, key genes were screened, and the KEGG pathway was enriched.

### 2.9. Real-Time Quantitative PCR

The RNA was extracted from liver tissue or cells using the SteadyPure Universal RNA Extraction Kit (Acres Bio, Hunan, China), while RNA concentration was measured using the NANO DROP 200 (Gene Company, China). One microgram RNA was reverse transcribed to cDNA using the EVO M-MLV Reverse Transcription Kit (Accurate Biotechnology Hunan, China). Quantitative RT-PCR was performed using the SYBR Green Pro Taq HS Premix qPCR kit (Accurate Biotechnology) on the Applied Biosystems StepOne Plus instrument (Thermo Fisher, USA). *β*-Actin was used as an internal control, and the 2^−ΔΔct^ method was applied to calculate mRNA gene expression levels. The primer sequences are shown in Table 1.

### 2.10. Western Blot Analysis

Liver tissues or cells were homogenized with RIPA lysis buffer and centrifuged at 12,000 rpm for 15 minutes before the supernatant was collected. Protein concentration was determined using the BCA protein assay kit (ComWin Biotech, Beijing). After 30 µg proteins were denatured by placing samples in an environment at 95°C for 5 minutes, proteins were then separated through 10% acrylamide gel electrophoresis and transferred to PVDF membranes (Millipore, Darmstadt, Germany). The membranes were incubated with primary antibodies at 4°C and left overnight. The next day, secondary anti-rabbit antibodies were added, and samples were incubated at room temperature for 1 hour. After incubation with ECL luminescent solution (Millipore), images were acquired using Tanon-5200 (Shanghai, China). ImageJ software was used to analyze the grayscale values of the strips, and *β*-actin was used as an internal loading control. Further information on the primary antibodies used in this study is shown in Table 2.

### 2.11. Cell Experiment

Alpha mouse liver 12 (AML12) cell lines were purchased from the Cell Biology Institute of Chinese Academy of Science (Shanghai, China) and cultured in DMEM/F12 with 10% FBS and 1% penicillin/streptomycin (Lonsera, Grand Island, USA) at 37°C in a humidified atmosphere containing 5% CO_2_. To induce hepatic steatosis model, cells were exposed to DMEM containing a 1 mM long-chain mixture of FFAs (oleate acid : palmitate acid = 2 : 1) and 1% BSA (Sigma, Steinheim, Germany) for 24 h. Three different doses of LZG, Fenofibrate (PPAR*α* agonist), or Gw6471 (PPAR*α* antagonist) (MedChemExpress, Shanghai, China) were added to the mixture of FFAs to incubate AML12 cells for 24 h, respectively. The doses of these medicines were determined according to cell viability tests for AML12 cells and/or the concentration for 50% of maximal effect (EC50). The cells cultured in the DMEM with 1% BSA were used as the control.

The cell viability of AML12 cells was measured using the Cell Counting Kit-8 (CCK-8, Dojindo, Kumamoto, Japan). The optical density (OD) of the cultures was measured at the absorbance of 450 nm by using Synergy H4 Hybrid Multi-Mode microplate reader (BioTeck, Winooski, USA). The lipid content in cells was determined using NileRed (SIGMA, Steinheim, Germany) and DAPI (MP, Biomedicals, USA) staining and scanned by ImageXpress Micro System (Molecular Devices).

### 2.12. Statistical Analysis

Data were expressed as mean ± standard deviation (mean ± SD). IBM SPSS Statistics 25 (SPSS Inc., Chicago, IL, USA) and GraphPad Prism 8.0 (GraphPad Software Inc., San Diego, CA, USA) were used to conduct statistical analysis of the data. Statistical analyses were carried out using *t*-test between two groups and one-way analysis of variance (ANOVA) followed by Tukey's post hoc comparison for three groups. *P* < 0.05 was considered a statistically significant difference.

## 3. Results

### 3.1. Lanzhang Granules Reduced Body Weight, Liver Weight, and Liver Index of NAFLD Mice

**Figure 1:**
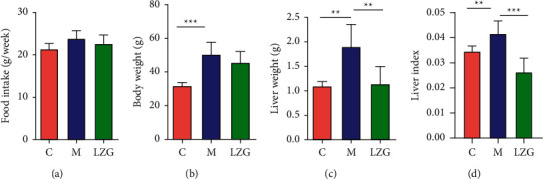
The effect of LZG on food intake, body weight, liver weight, and liver index of NAFLD mice. (a) Food intake weekly. (b) Body weight. (c) Liver weight. (d) Liver index. *n* = 6; ^*∗∗*^*P* < 0.01; ^*∗∗∗*^*P* < 0.001.

There was no significant difference in weekly food intake among the three groups of mice. Compared with the normal group, the body weight, liver weight, and liver index (liver-to-body ratio) of mice in the high-fat-fed NAFLD model group were significantly increased. LZG intervention for 8 weeks significantly reduced the liver weight and index and showed decreased trend of body weight in mice (Figure 1).

### 3.2. Lanzhang Granules Attenuated the Level of Serum Transaminases, LDH, Lipids, and Inflammatory Factors in NAFLD Mice

**Figure 2:**
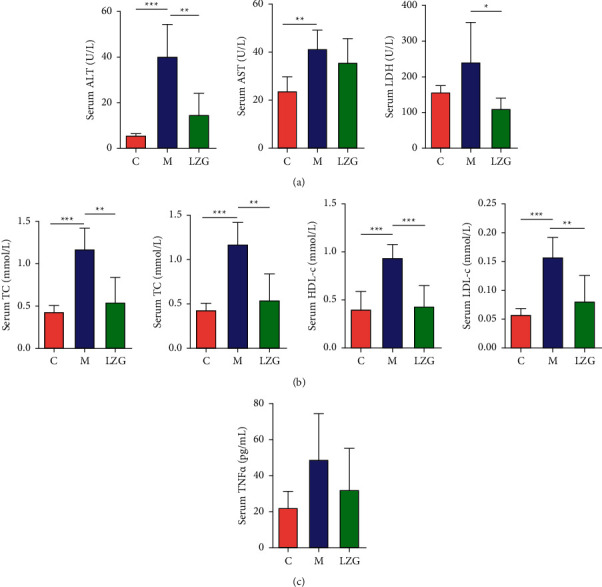
The effect of LZG on serum biochemical indices and proinflammatory factor levels in NAFLD mice. (a) Serum ALT, AST, and LDH. (b) Serum TG, TC, HDL-c, and LDL-c. (c) Serum TNF*α* levels in mice. *n* = 6; ^*∗*^*P* < 0.05; ^*∗∗*^*P* < 0.01; ^*∗∗∗*^*P* < 0.001.

ALT and AST are important indicators of liver injury. HFD caused elevated serum ALT, AST, and LDH in mice, while LZG significantly reduced serum ALT and LDH. LZG also had a tendency to downregulate AST (Figure 2(a)). In addition, LZG was able to downregulate the elevated levels of serum TC, HDL-c, and LDL-c in NAFLD mice induced by the high-fat diet. There was no significant change in serum TG levels in any of the groups (Figure 2(b)). Researchers also observed a trend of upregulation of serum TNF*α* in the model group, along with downregulation of serum TNF*α* in the LZG group in mice (Figure 2(c)).

### 3.3. Lanzhang Granules Improved Liver Histopathology and Liver Lipid Levels in NAFLD Mice

**Figure 3:**
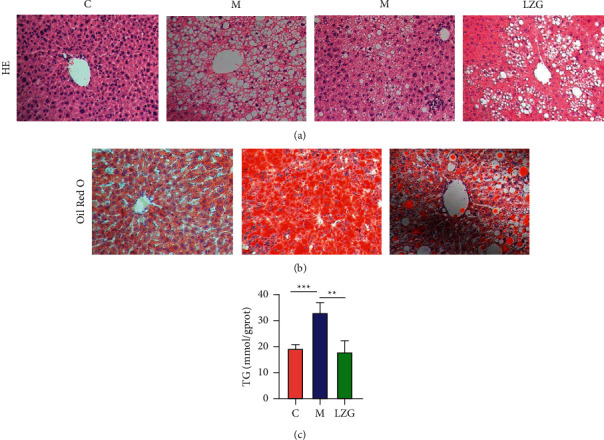
LZG improved liver histopathology and decreased liver TG level in NAFLD mice. (a) HE staining of liver tissues (×200). (b) Oil Red O staining of frozen sections of liver tissues (×200). (c) TG level of liver tissues in mice. *n* = 6; ^*∗∗*^*P* < 0.01; ^*∗∗∗*^*P* < 0.001.

In the liver tissues of HFD-induced NAFLD model mice, HE staining and Oil Red O staining of liver tissues showed a large number of lipid droplets, indicating diffuse steatosis in the liver. In addition, focal inflammatory infiltration was found in the liver tissue of model mice. LZG was able to effectively reduce the lipid aggregation and inflammation in liver tissues (Figures 3(a) and 3(b)). The TG content in the liver of mice in different groups is reflected in Figure 3(c). These results also indicated that LZG could alleviate lipid accumulation.

### 3.4. Lanzhang Granules Improved Insulin Resistance in NAFLD Mice

**Figure 4:**
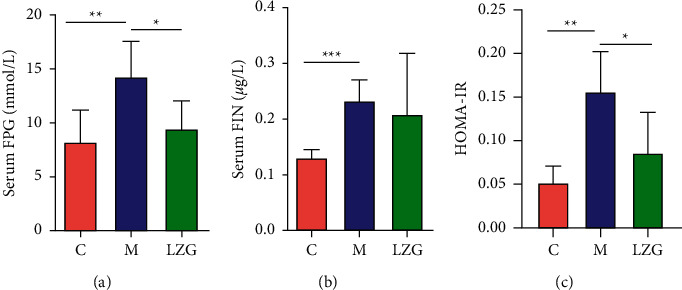
The effect of LZG on insulin resistance in NAFLD mice. (a) Fasting serum glucose levels in mice. (b) Fasting insulin levels in mice. (c) Serum HOMA-IR values in mice. *n* = 5; ^*∗*^*P* < 0.05; ^*∗∗*^*P* < 0.001; ^*∗∗∗*^*P* < 0.0001.

The levels of fasting glucose (FPG) and fasting insulin (FIN) were significantly higher in NAFLD mice compared to controls. The administration of LZG resulted in an effective reduction in FPG levels, but a downregulation of FIN levels. The HOMA-IR values were significantly higher in the model mice than in the control while being downregulated by the LZG treatment (Figure 4). These results suggest that LZG could improve insulin resistance in NAFLD mice.

### 3.5. Differential Genes Screened by RNA Sequence and Validation

**Figure 5:**
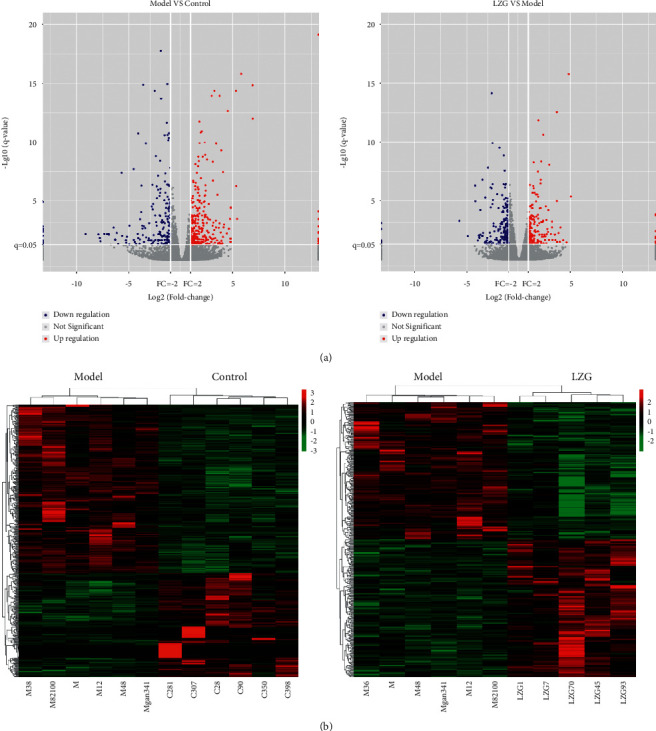
Volcano and heat map of differentially expressed genes. (a) Volcanic map of differentially expressed genes. (b) Heat map of differentially expressed genes.

In order to further clarify the mechanism and action of LZG, we performed gene RNA sequencing of liver tissues. The volcano map and heat map showed that there were a large number of differential genes between the control, model, and LZG groups. There were 78 differential genes present in the model group when compared to the control group, including 48 upregulated genes and 30 downregulated genes. When compared with the model group, the LZG group had 277 differential genes, including 141 upregulated genes and 136 downregulated genes (Figure 5).

**Figure 6:**
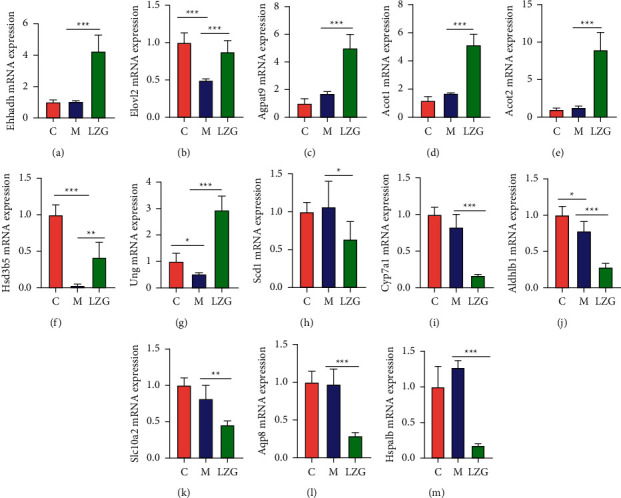
Differentially expressed genes in liver tissues of mice in the LZG group and model group. (a–g) Upregulated gene mRNA expression levels in the LZG group as compared to the model group. (h–m) Downregulated gene mRNA expression levels in the LZG group as compared to the model group. *n* = 6; ^*∗*^*P* < 0.05; ^*∗∗*^*P* < 0.01; ^*∗∗∗*^*P* < 0.001.

In order to make the gene expression levels comparable across genes and samples, we converted the reads into fragments per kilobase of exon model per million mapped reads (FPKM) for normalization of gene expression. We screened 13 differential genes between the model group and the LZG group according to *q* value ≤0.05 and fold change ≥2 (Table 3). Among these, there were six downregulated genes and seven upregulated genes observed in the LZG group as compared to the model group. This 13 gene expression was further validated by qRT-PCR. Similar results were found with the RNA sequence (Figure 6).

### 3.6. Analysis of Differential Signaling Pathways Reveals Modulation of PPAR*α* Pathway by Lanzhang Granules and Validation

**Figure 7:**
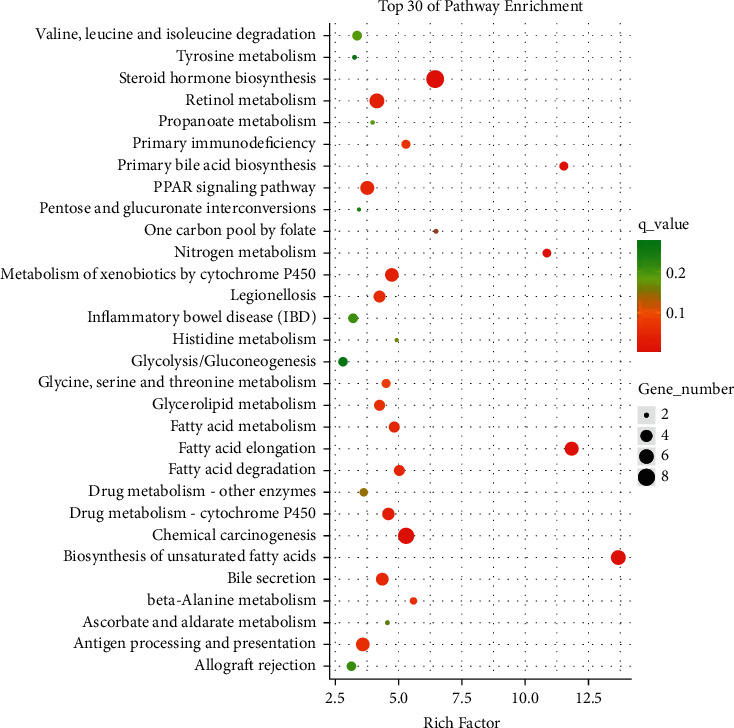
KEGG enrichment scatter plot of differentially expressed genes in the LZG group versus the model group (top 30 pathways).

**Figure 8:**
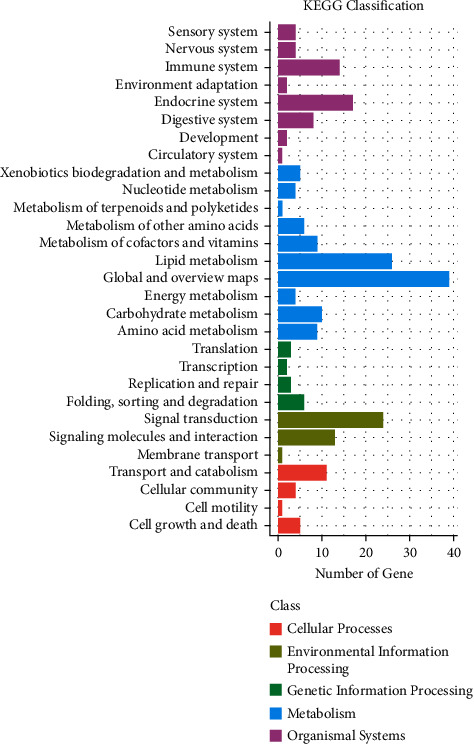
Statistical map of KEGG pathway classification of differentially expressed genes between the LZG group and the model group.

Subsequently, we performed KEGG enrichment of differential genes between the LZG and model groups to screen for and classify the TOP30 pathways (Figures 7 and 8). This helped to identify the key pathways regulated by LZG. KEGG analysis revealed that the major differential signaling pathways included steroid hormone biosynthesis, the peroxisome proliferator-activated receptor (PPAR) signaling pathway, primary bile acid biosynthesis, and the biosynthesis of unsaturated fatty acids. The classification of the pathways showed that the differential signaling pathways focused on the regulation of lipid metabolism.

**Figure 9:**
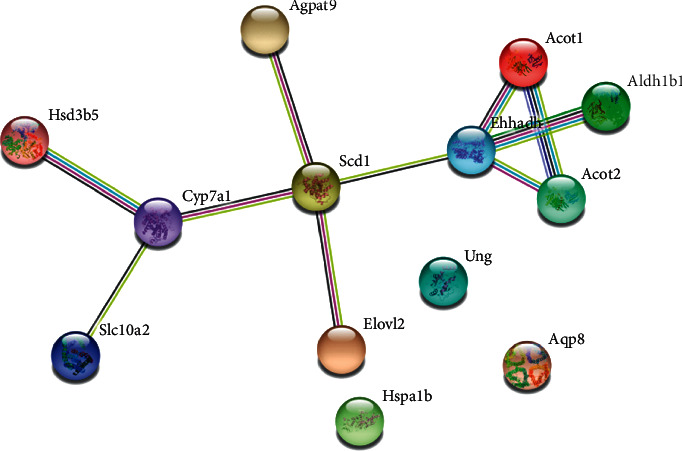
The interaction of differential proteins between the LZG group and the model group (PPI database).

Based on the 13 differential genes between the model group and the LZG group, the PPI database was applied to enrich the differential gene-related KEGG pathway (Table 4). The results showed that these differential genes were mainly involved in lipid metabolism-related signaling pathways, including the PPAR pathway, which is closely related to metabolism. Interaction analysis (PPI database) of 13 differential gene-expressed proteins (Figure 9) suggested that peroxisomal L-bifunctional enzyme (EHHADH), cholesterol 7 alpha-hydroxylase (CYP7A1), and stearoyl-CoA desaturase 1 (SCD1) are the key regulatory genes. All of these are key molecules involved in the PPAR*α* signaling pathway. Therefore, it is hypothesized that the PPAR*α* signaling pathway may be one of the pivotal mechanisms by which LZG exerts its effects on NAFLD.

**Figure 10:**
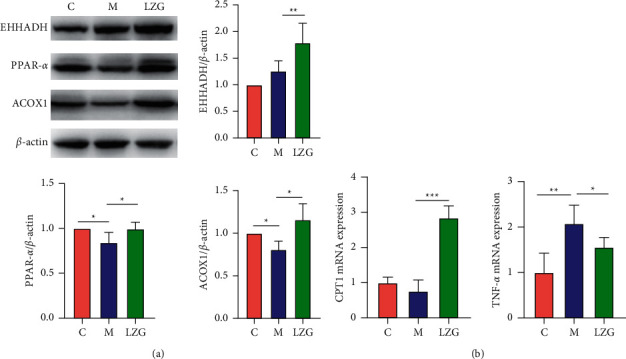
The effect of LZG on PPAR*α* pathway-related protein and gene expression in NAFLD mice. (a) Protein expression levels of EHHADH, PPAR*α*, and ACOX1 in the liver tissues of mice. (b) The relative mRNA expression of CPT1 and TNF*α* in the liver tissues of mice. *n* = 6; ^*∗*^*P* < 0.05; ^*∗∗*^*P* < 0.01; ^*∗∗∗*^*P* < 0.001.

The protein immunoblotting assay was applied to further detect the protein levels of EHHADH in mouse liver tissues (Figure 10(a)). The results showed that EHHADH protein expression was upregulated by LZG, which was consistent with the results of previous analyses. To further clarify the regulatory effect of the drug on the PPAR*α* pathway, the expression levels of PPAR*α* and its target genes lipid oxidation gene acyl-coenzyme A oxidase 1 (ACOX1), carnitine palmitoyltransferase1 (CPT1), and TNF*α* were examined. It was found that LZG upregulated the expression levels of PPAR*α* protein, which was reduced in model mice. The results showed a decrease or downregulated tendency of ACOX1 and CPT1 in the model group. And there was a significant upregulation in TNF*α* expression when compared to the control mice (Figure 10(b)). LZG could upregulate the expression level of these two gene expressions and improve the expression of TNF*α* induced by the high-fat diet in the model group.

### 3.7. Lanzhang Granules Improved the Lipid Accumulation, Cell Injury, and PPAR*α*-Regulated Gene Expression in AML12 Cells Induced by Fatty Acid

**Figure 11:**
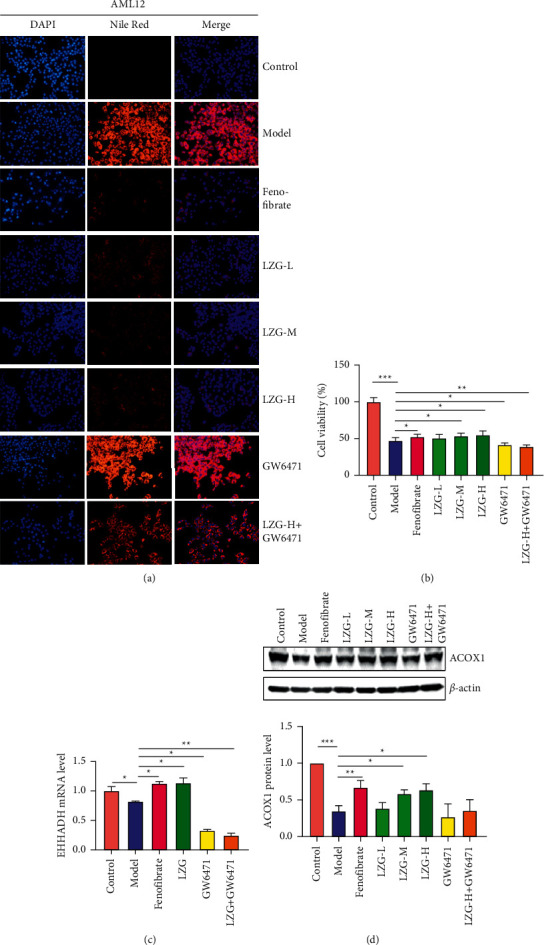
The effect of LZG on lipid accumulation, cell injury, and PPAR*α*-regulated gene expression in AML12 cells induced by fatty acid. (a) The lipid accumulation indicated by Nile red staining. (b) The cell viability. (c) The mRNA expression of EHHADH relative to *β*-actin. (d) Protein expression of ACOX1 relative to *β*-actin. *n* = 3; ^*∗*^*P* < 0.05; ^*∗∗*^*P* < 0.01; ^*∗∗∗*^*P* < 0.001.

To clarify the effect of LZG on NAFLD through regulating PPAR*α* signaling, *in vitro* experiments were carried out by using AML12 cells induced by fatty acids as a NAFLD *in vitro* model. The results showed that LZG and PPAR*α* agonists Fenofibrate upregulated the expression level of PPAR*α*-targeted genes ACOX1 and EHHADH and improved lipid accumulation as well as cell viability, while PPAR*α* antagonist GW6471 had the opposite effect. Coincubation with GW6471 blocked LZG's regulation of PPAR*α* target gene transcription and the improvement of lipid accumulation and cell viability (Figure 11).

## 4. Discussion

The characteristics of the NAFLD model induced by a high-fat diet include weight gain, liver damage, lipid accumulation, and insulin resistance. These effects are similar to the pathological process of human NAFLD. In this study, after high-fat diet induction for 30 weeks, the model mice showed obesity, liver steatosis, and elevated ALT. Insulin resistance was found to be an independent predictor of NAFLD [8], and the HOMA-IR index was found to be significantly increased in the high-fat-induced model group of mice in this study. These results are consistent with what has been demonstrated by previous studies [9]. Intervention with Lanzhang Granules for 8 weeks significantly reduced body weight, liver weight, and liver index. In addition, this treatment also downregulated serum ALT, AST, and LDH levels. The changes of serum LDL-c, TC, and TG in mice indicated that LZG could improve blood lipid levels. The HE staining, Oil Red O staining, and TG level of mouse liver tissues showed that Lanzhang Granules significantly reduced hepatic steatosis. In addition, we found that LZG could downregulate insulin resistance in NAFLD mice by calculating HOMA-IR values. These results confirmed that LZG could effectively improve NAFLD in mice.

In order to further investigate the efficacy mechanism of LZG on NAFLD, the liver tissues of mice were examined by RNA sequencing to screen the differential genes between the model and LZG groups. The 13 genes with the most significant differences were screened and validated. By enriching the KEGG pathway and analyzing the interactions between the 13 differential genes proteins using PPI, it was tentatively concluded that LZG improved liver steatosis in mice, mainly through the PPAR*α* pathway.

PPAR*α* is a metabolism-related nuclear receptor, acting as a nuclear transcription factor that regulates lipid homeostasis in the body. It is present in tissues with high metabolic rats, such as skeletal muscle, heart, liver, and brown fat [10]. It can regulate the expression of genes related to fatty acid uptake, intracellular transport, fatty acid activation, fatty acid oxidation, adipogenesis, ketogenesis, and lipoprotein/cholesterol metabolism [11, 12]. PPAR*α* is strongly associated with NAFLD [13]. The deletion of PPAR*α* was found to promote obesity and steatosis induced by HFD in PPAR*α* knockout mice as compared to the wild-type mice [14]. In addition, PPAR*α* agonists have been proved to reduce triglycerides and free fatty acids [15]. In humans, PPAR*α* expression level is negatively correlated with the disease severity of NASH [16]. Clinical studies have shown that the increased expression of PPAR*α* in the human liver can alleviate visceral obesity and insulin resistance while improving the effect of histology [17]. The current studies found that PPAR*α* level reduced in the model mice, while it was increased by LZG treatment. This suggests PPAR*α* may be the potential therapeutic target of LZG.

Sustained lipid accumulation initiates pathological stages of NAFLD. Studies have revealed that PPAR*α* serves as the master regulator of liver lipid metabolism. PPAR*α* activity for using fatty acid is important to block steatosis during fasting in mice [18]. PPAR*α* is involved in the regulation of peroxisomal and mitochondrial *β*-oxidation, as well as microsomal *ω*-oxidation [19]. PPAR*α* regulates the expression levels of the rate-limiting enzymes of peroxisomal *β*-oxidation, including ACOX1 and EHHADH [12]. EHHADH can also induce the activation of PPAR*α* and ACOX1 [20]. It has previously been shown that PPAR*α* can regulate CPT levels, which transport the fatty acid across the mitochondrial membrane [21]. The activation of PPAR*α* can promote the migration of fatty acids to mitochondria [22]. Our results showed that LZG treatment obviously increased the expression of EHHADH, ACOX1, and CPT1 in the liver tissues of NAFLD mice and AML12 cells with steatosis. Coincubation with PPAR*α* antagonist reversed the regulation in cells. This suggests that LZG could alleviate hepatic steatosis by regulating PPAR*α* signaling to promote *β*-oxidation of fatty acid.

Chronic inflammation is also one frequent feature of NAFLD. It has been found that elevated levels of TNF*α* expression activate IкB kinase *β*, inhibit insulin receptor substrates 1 and 2, and activate fatty acid translocase (FAT) in the liver. FAT promotes the uptake of long-chain fatty acids (LCFA) from the blood by the liver and exacerbates liver lipid deposition [23]. In addition to regulating metabolism, PPAR*α* negatively regulates the proinflammatory signaling pathway via protein-protein interactions or PPRE-dependent transcriptional repression [12]. Previous studies have shown that it can inhibit the transcription of proinflammatory mediators through complex regulation of transcription factor NF-*κ*B [24]. PPAR*α* knockout mice fed an HFD showed increased inflammatory markers [15]. In the present study, there was a decreasing trend of serum TNF*α* and an obvious downregulation of TNF*α* in mouse liver tissues in the LZG group as compared with the model group. With this in mind, it was suggested that Lanzhang Granules might also reduce liver inflammation and prevent NASH in mice by regulating PPAR*α*.

## 5. Conclusions

Lanzhang Granules can effectively improve the pathological changes and insulin resistance of NAFLD in mice. The mechanism of action may be through regulating the PPAR*α* pathway to improve the lipid metabolism and inflammation of NAFLD. In future studies, we will further clarify the detailed regulation of the relevant signaling pathways through *in vivo* and *in vitro* experiments. The study will help reveal the efficacy mechanism of Lanzhang Granules and promote their clinical application.

## Figures and Tables

**Table 1:** PCR primer sequences.

Species	Gene	Primer sequence (5'-3')
Mouse	Actin	Forward: GAGACCTTCAACACCCCAGC
		Reverse: ATGTCACGCACGATTTCCC
Mouse	Scd1	Forward: ATTGCCTATGAACTCAACAGCG
		Reverse: TGCCATAGTGGTTGAGGTTGG
Mouse	CYP7A1	Forward: TTCAAGACCGCACATAAAGCC
		Reverse: GAGATGCCCAGAGGATCACG
Mouse	Aldh1b1	Forward: CGGGCTCCACTGAGGTAGG
		Reverse: TGAAGAAAAGGGCTTCGTGAC
Mouse	SBTR	Forward: ATGGCGACATGGACCTCAG
		Reverse: TCCCGAGTCAACCCACATC
Mouse	Aqp8	Forward: AGCTATTTGGAGGGCTGATTG
		Reverse: CTATCCCCAGGGCTTCTGC
Mouse	HSP70	Forward: GAGGGTCTCAAGGGCAAGC
		Reverse: ACCCTGGTACAGCCCACTGA
Mouse	EHHADH	Forward: ATTGGAGTCGTTGTTGGTAACTG
		Reverse: CATCCTCTGGCTTACTACCTTCC
Mouse	Elovl2	Forward: CTCACCTTGTATAACCTCGCAATC
		Reverse: TCGTGTCCAGGAACTCCACTAG
Mouse	Acot1	Forward: ACCCTTTCCTGGGATCATAGAC
		Reverse: TTGGGGAGGTCATCGTAGTTG
Mouse	Acot2	Forward: CTCTGGCTTATTACAACTACGACG
		Reverse: CCCAATTCCAGGTCCTTTTAC
Mouse	Agpat9	Forward: CTATCTGGCACCATCCATTACC
		Reverse: CCATCAATCCACCGTGAACC
Mouse	Hsd3b5	Forward: AGCAAAAGGATGGCTGAGAAG
		Reverse: CATACACTGGGTTGGCGATG
Mouse	Ung	Forward: GCTGAAGAAAGGAACCACCAC
		Reverse: CTGAAGCAGAGCCCGTGAG
Mouse	PPAR*α*	Forward: CCTGAAAGATTCGGAAACTGC
		Reverse: GACAAAAGGCGGGTTGTTG
Mouse	TNF*α*	Forward: CCCTCCAGAAAAGACACCATG
		Reverse: CACCCCGAAGTTCAGTAGACAG
Mouse	Cpt1a	Forward: GCCATACTGCTGTATCGTCGC
		Reverse: CGGGAAGTATTGAAGAGTCGC

**Table 2:** The primary antibodies for Western blot.

Antibody	Company	CAT	Source	MW(KDa)	Dilution
*β*-Actin	CST	4970	Rabbit	42	1 : 1000
ENHADH	Abclonal	A13488	Rabbit	79	1 : 500
ACOX1	Proteintech	10957-1-AP	Rabbit	74 (50)	1 : 1000
PPAR*α*	Proteintech	15540-1-AP	Rabbit	52	1 : 500

**Table 3:** Differentially expressed genes in liver tissues of mice between the LZG group and model group.

Genus	Gene ID	Description	Gene names	Pathways	Trend
Mouse	ENSMUSG 00000037071	Stearoyl-coenzyme A desaturase 1	Scd1	mmu04152|mmu03320|mmu01212|mmu01040	Down
Mouse	ENSMUSG 00000028240	Cytochrome P450 family 7 subfamily A polypeptide 1	Cyp7a1	mmu01100|mmu00140|mmu03320|mmu04976	Down
Mouse	ENSMUSG 00000035561	Aldehyde dehydrogenase 1 family member B1	Aldh1b1	mmu01100|mmu00010|mmu00561|mmu00280|mmu00310|mmu00071|mmu00330|mmu00380|mmu00620|mmu00040|mmu00410|mmu00053|mmu00340	Down
Mouse	ENSMUSG 00000023073	Solute carrier family 10 member 2	Slc10a2	mmu04976	Down
Mouse	ENSMUSG 00000030762	Aquaporin 8	Aqp8	mmu04976	Down
Mouse	ENSMUSG 00000090877	Heat shock protein 1B	Hspa1b	mmu04144|mmu04010|mmu05169|mmu05164|mmu04141|mmu05162|mmu03040|mmu05145|mmu04915|mmu04612|mmu04213|mmu05134	Down
Mouse	ENSMUSG 00000022853	Enoyl-coenzyme A hydratase and 3 hydroxyacyl coenzyme A dehydrogenase	EHHADH	mmu01100|mmu01200|mmu04146|mmu03320|mmu00280|mmu00310|mmu01212|mmu00071|mmu00380|mmu00410|mmu00640|mmu00650	Up
Mouse	ENSMUSG 00000021364	Elongation of very long-chain fatty acids FEN1 Elo2 SUR4 Elo3 yeast like 2	Elovl2	mmu01212|mmu01040| mmu00062	Up
Mouse	ENSMUSG 00000072949	Acyl-CoA thioesterase 1	Acot1	mmu01100|mmu01040|mmu00062	Up
Mouse	ENSMUSG 00000021226	Acyl-CoA thioesterase 2	Acot2	mmu01100|mmu04913|mmu01040|mmu00062	Up
Mouse	ENSMUSG 00000029314	1-Acylglycerol-3-phosphate O acyltransferase 9	Agpat9	mmu01100|mmu00564|mmu00561	Up
Mouse	ENSMUSG 00000038092	Hydroxy-delta-5-steroid dehydrogenase 3 beta and steroid delta isomerase 5	Hsd3b5	mmu01100|mmu00140|mmu04925|mmu04913	Up
Mouse	ENSMUSG 00000029591	Uracil DNA glycosylase	Ung	mmu03410|mmu05340	Up

**Table 4:** Differential gene KEGG pathway between LZG group and model group (PPI database).

Pathway	Description	Count in network	Strength	False discovery rate
mmu01040	Biosynthesis of unsaturated fatty acids	4 of 27	2.4	9.94*e* − 08
mmu00062	Fatty acid elongation	3 of 26	2.29	1.27*e* − 05
mmu00410	Beta-alanine metabolism	2 of 32	2.03	0.0011
mmu01212	Fatty acid metabolism	3 of 51	2.0	4.53*e* − 05
mmu00380	Tryptophan metabolism	2 of 45	1.88	0.0019
mmu04976	Bile secretion	3 of 71	1.86	8.90*e* − 05
mmu00071	Fatty acid degradation	2 of 50	1.83	0.0020
mmu00280	Valine, leucine, and isoleucine degradation	2 of 55	1.79	0.0022
mmu03320	PPAR signaling pathway	3 of 85	1.78	0.00012
mmu04913	Ovarian steroidogenesis	2 of 57	1.77	0.0022
mmu01212	Lysine degradation	2 of 58	1.77	0.0022
mmu01212	Glycerolipid metabolism	2 of 60	1.75	0.0022
mmu01212	Steroid hormone biosynthesis	2 of 83	1.61	0.0035
mmu01212	Metabolic pathways	7 of 1296	0.96	4.53*e* − 05

## Data Availability

The datasets used to support the findings in the current study are included in the article.
